# The Present Status of Obstetrics and Gynæcology*Read before the Ohio State Medical Society, in Cincinnati, May, 1892.

**Published:** 1892-06

**Authors:** Chauncey D. Palmer

**Affiliations:** Cincinnati, Ohio


### Southern Medical Record.

A MONTHLY JOURNAL OF MEDICINE AND SURGERY.

VOL. XXII. ATLANTA, GA., JUNE, 1892. No. 6

### Original Articles.

### THE PRESENT STATUS OF OBSTETRICS AND GYNÆCOLOGY.*

BY CHAUNCEY D. PALMER, M. D , CINCINNATI, OHIO.

In 1878, I delivered the annual address of the Alumnial Association of the Medical College of Ohio, on the subject of "The Present Status of Gynæcology and its Relations to General Medicine." This was nearly fifteen years since. It has occurred to me that there is no subject fcr me better adapted to a society of this kind, composed of gentlemen of almost all ages and specialties of medicine, of the great State of Ohio, than this very same one.

What is the present status of obstetrics and gynæcology to-day? No one will for a moment say that it is what it was fifteen years since. What it will be fifteen years from now, is only a matter of speculation.

To the thoughtful inquirer, it is plain that the relation of obstetrics and gynæcology to general medicine is a most intimate and inseparable one. The idea that a physician may practice gynæcology in its exclusive sense, without a knowledge of the diseases of the body at large, or with a

*Read before the Ohio State Medical Society, in Cincinnati, May, 1892.

disregard of the general bodily conditions, is one of the most absurb and dangerous doctrines. There is comparatively little gynæcology outside of the domain of general medicine; on the other hand, there is scarcely a disease of the general system in the female, of any considerable importance or duration, which does not affect the circulation, innervation, and functions of the pelvic organs. We venture the assertion, and challenge its successful contradiction, that no disease or class of diseases in the economy, set up more decided disturbances or complications at large than those of the female pelvic organs.

Medicine, although made up of parts, is really one. The whole stands by a linking together of all its parts; a separation of any link implies a break in the whole chain. Gynæcology has suffered not a little by this *exclusiviem* of our times. The extreme surgical tendency of some, has brought us an unmerited disrepute. Pelvic diseases must be learned by studying medicine in its entirety. Few constitutional diseases, or even local affections of distant organs of the body exist, that we do not see in women some peculiar expression of the same in the sexual system. The various states of a changed constitution of the blood, the so-called diathesis and cachexiæ, as anæmia, hydræmia, leucocythæmia, scrofula, phthisis, rheumatism, gout, syphilis, scorbutus, eczema, chronic malaria, almost invariably, unfortunately too rapidly, first disorder menstruation, and finally produce organic changes in the uterus. A neurotic habit, evidenced by attacks of general neuralgia, is accompanied by a neuralgic dysmenorrhœa, as is also such a constitution a very important factor in the causation of other pelvic affections.

Again pregnancy, more particularly the lying-in state, tests the soundness of a woman's constitution. Any imperfection of her system, any blood taint, although hidden, is apt to manifest itself at this time. Pelvic cellulitis, we have noticed, has at times an etiological constitutional factor. So has mammary inflammations, an enfeebled general health, a disease prior to marriage, present though latent, frequent child-bearing, prolonged and excessive lactation, and many other general causes make a woman liable to and perpetuate sub-involution, hyperæmia of the uterus, with displacements, diseases and complications from which she would otherwise be exempt. Many of the well-known exciting causes utterly fail to bring about a local disease, without some constitutional morbific force operative in the background. In this way, a discovery of one's actual constitutional standard of health is revealed. Many of the chronic diseases of the uterus are chronic only in virtue of a diathetic taint—a general imperfection or depreciation of the general health. Therefore, that local diseases of the female pelvic organs require only a local treatment, is a pernicious doctrine, wrong in theory, dangerous and criminal in practice. A gynæcologist cannot be a skillful physician without a knowledge of medicine in general. It is necessary that he diagnosticate, know the causes and proper management of diseases in general. Constantly arising in his special practice, they influence and complicate the acute and chronic conditions for which his services are sought. In turn, while it is not to be expected of the general practitioner that he will differentiate uncommon and obscure pelvic affections, or perform special operations in this field of practice, it is expected that he will understand the importance and significance of symptoms here arising, and the general management of the diseases they express.

Volumes might be written on the influences of civilization and modern culture, with its fashionable surroundings and entanglements, modifying the diseases of women. Unquestionably, both sexes do not have a few of the physical ailments of a generation since, but it remains a seriously debatable question whether women now-days do not have many of their diseases more frequently. I believe they do, and look upon it as a penalty of our modern methods of living.

Local medication of the uterus is certainly better understood now than it was fifteen years since. There can be no question as to the propriety of local treatments, but an ailing woman, afflicted with a disease of her sex, must be at the mercy of the good judgment of an honest physician.

*When* to medicate, *when not* to medicate the uterus, *how*

*often* and *with what*, are questions to be answered by a studious, progressive practitioner.

Fluid applications to the uterus have now a diminished field of utility. Intra-uterine medication can be made less painful, less hazardous, and more efficient. Potential cauterization of the uterus is now much less frequently employed, and the use of the inevitable *nitrate* has almost become a relic of the past. Who can doubt that we better know how to treat endometrial affections than we did? That the endometrium is rather a glandular than a mucous tissue, is recognized.

Gonnorrhœa, it matters not how contracted, as a causative factor in many female pelvic diseases, has a hitherto unrecognized significance. Massage, as a therapeutic agent in gynæcology, is well appreciated, to say naught about the local massage after *thure brandt*. Electricity as a therapeutic agent, never received such attention. *Apostoli* has been, and is an enthusiastic, painstaking, and progressive worker. His enunciations have been seconded by careful, observing men all over the world, and he is considered to be not altogether a misguiding leader. Competent judges in gynæcology say we must use careful judgment and fair discretion in the selection of cases, *in kind*, if we do good with electricity for fibroids of the uterus. There is really no contention between electricity and surgery, in gynæcology. Each has its place and field of application, hence, usefulness. The *Apostoli* treatment for uterine fibroids, embraces by no means all the good we have in this line of practice. Unmistakable benefits accrue from electrical treatments in certain conditions of chronic pelvic inflammatory exudations, uterine versions and flexions, as well as for some of the various forms of uterine inflammations. We know comparatively little more concerning pelvic cellulitis and pelvic peritonitis than have been so clearly and reliably announced by *Bernutz* and *Goupil* in 1857. But our acquaintance with the diagnosis and operative cure of diseases of Fallopian tubes, is only of rather recent date. The gynæcological world had its craze of cervical splitting, then its trachelorraphy craze, its pessary craze, and now it is having its Fallopian craze.

A more clear and thorough elucidation of the diagnostic signs of pregnancy, intra and extra, have occurred in recent years. A recognition of early pregnancy depends, usually, not on one symptom or group of symptoms, but on a painstaking analysis of the sum total of evidences in a given case. The most conclusive evidence is found within the pelvis, in the size, shape and consistency of the uterus and surroundings, no sign, but the collective evidences of all signs, being considered. Many mistakes in diagnosis of pregnancy are avoidably and unavoidably made. It is well always to suspect for ourselves the possible existence or co-existence of pregnancy in any female whom we are examining, if between ten and fifty-five.

One of the most important advances of medical and surgical gynæcology has been the recognition of bacteria in producing certain morbid processes. How many cases of endometritis and salpingitis we now know are septic in their origin? Consider the diminished and diminishing mortality rates in modern obstetric practice. One death in one-thousand cases of parturition in Paris, and none in as many cases in Preston Retreat, would have astonished our predecessors. So much for improved aseptic and antiseptic precautions! We have come to understand that sepsis, not traumatism, is the danger in all obstetrical and gynæcological operations. We know now how to employ the uterine curette, a most valuable instrument, calculated to supercede much intra-uterine medication, so that its ills may be reduced to a minimum. Pessaries, we are not, as yet, ready to dispense with, although their use has been judiciously, greatly abridged. Some hitherto regarded incurable uterine displacements have been made amenable to medical and surgical treatment. Certain surgical affections of women, as vesico-and recto-vaginal fistulæ, lacerations of the cervix uteri and vagina, and perineum, are recognized as preventable diseases, and their best treatments are prophylactic and immediate. Stitch-hole abcesses, abdominal herniæ and fæcal fistulæ can generally be avoided. The greatest improvements in modern surgical gynæcology and obstetrics, prophylactic, plastic and abdominal, we owe to *Semmelweis* and *Lister*. A greater revolution in political history, anywhere, at any time, never occurred, than these men have been the agents of originating in surgery.

The better methods of perineal repair, together with all the plastic operations to correct the injuries of pelvic structures from parturition, have been developed, if not perfected, within a few years.

In no direction has gynæcology grown in recent years, as in the surgical. Compare the most excellent work of *Greig Smith* of to-day with the gynæcology in surgery of 1856.

In 1855, *Spencer Wells* published a small book on the diagnosis and surgical treatment of abdominal tumors. "Is ovariotomy justifiable?" was the question then. Now everyone advocates its justifiability, and early. In recognition of the grand work which has been done for this otherwise incurable disease, witness the operations of the liver, gall bladder, stomach, spleen, kidneys, intestines and other abdominal and and pelvic structures. Section, peritoneal irrigation, and drainage, have revolutionized abdominal and pelvic surgery. It is impossible to practice gynæcology without a resort to surgical procedures. Such is the causation and nature of many of the gynæcological diseases that surgical operations for them is a *sine qua non.* Modern gynæcology has made its most wonderful advance by surgery. See what it has done for extra-uterine gestation, uterine cancer, some kinds of uterine displacements, and otherwise unrelieved uterine fibroids. Not only has vaginal hysterectomy established for it a proper place, but through supra-vaginal hysterectomy, is destined to be the operation, if any, for uterine contain, not all uterine fibroids. No real contest between high amputation and complete vaginal hysterectomy for uterine cancer exists.

More than my allotted time has been consumed in praise of what gynæcology *has* done and *will* do; and many pages might be written concerning modern gynæcological abuses. Every department of medicine and surgery has been subject to abuses. Intra-uterine medications, the *Emmett* operation of trachelorraphy, the *Sims*' operation of cervical incisions, the *Battey's* operation of oophorectomy, and not a few operations by laparotomy, and vaginal hysterectomy, have each and all been abused. No intelligent, rational physician will, for a moment, attempt to assert that these surgical procedures have no place in practice, and should be ignored. It would be well if every medical man would carefully consider the supposed curative effect of operations *per se.* Medical records are full of them. The physical influences at work are, at times, a most interesting study. The influences of the body, especially the pelvic organs of females, on the mind, are ever to be held in view. We should ever endeavor to avoid developing a neurosis, through intra-spection, especially in mobile patients. I would discountenance the removal of the uterine appendages for mental disturbances of any kind, for all cases of hysteria, most cases of hystero-epilepsy, dysmenorrhœa, and many cases of neurotic pelvic pains; at least, they should be done if at all only as a last resort, and only after full consent and explanation to patient and her family of results expected.

The modern tendency of to-day is to make many of the female pelvic operations on a very small pretext—made unnecessarily, it may be, either for life, comfort, or the preservation of health. Many of the well recognized failures of oophorectomy and salpingo-oophorectomy have followed operations done for hystero-neuroses, under a mistaken diagnosis.

Notwithstanding all the draw-backs and abuses gynæcology has encountered, the young science and art applied have relieved many a useful life and saved from the grave many a worthy, noble companion for life, otherwise unhelped.

